# Epidemiology of heart failure in Türkiye

**DOI:** 10.55730/1300-0144.5930

**Published:** 2024-05-07

**Authors:** Naim ATA, İnci Tuğçe ÇÖLLÜOĞLU, Anıl ŞAHİN, Mehmet Birhan YILMAZ, Sanem NALBANTGİL, Şuayip BİRİNCİ, Mustafa Mahir ÜLGÜ, Emine Arzu KANIK, Dilek URAL, Lale Dinç ASARCIKLI, Emre DEMİR, Yüksel ÇAVUŞOĞLU, Selda MURAT, Ahmet ÇELİK

**Affiliations:** 1 General Directorate of the Health Information Systems, Ministry of Health, Ankara, Turkiye; 2 Department of Cardiology, Faculty of Medicine, Karabük University, Karabük, Turkiye; 3 Department of Cardiology, Faculty of Medicine, Sivas Cumhuriyet University, Sivas, Turkiye; 4 Department of Cardiology, Faculty of Medicine, Dokuz Eylül University, İzmir, Turkiye; 5 Department of Cardiology, Faculty of Medicine, Ege University, İzmir, Turkiye; 6 Deputy Health Minister, Ministry of Health, Ankara, Turkiye; 7 Department of Biostatistics, Faculty of Medicine, Mersin University, Mersin, Turkiye; 8 Department of Cardiology, Faculty of Medicine, Koç University, İstanbul, Turkiye; 9 Department of Cardiology, Faculty of Medicine, Health Sciences University, İstanbul, Turkiye; 10 Department of Cardiology, Faculty of Medicine, Eskişehir Osmangazi University, Eskişehir, Turkiye; 11 Department of Cardiology, Faculty of Medicine, Mersin University, Mersin, Turkiye

**Keywords:** Heart failure, epidemiology, regional variations, pediatric population, incidence, prevalence

## Abstract

**Background/aim:**

The epidemiological data on heart failure (HF) vary between regions within the same country. We aimed to investigate the epidemiological data on HF in Türkiye across all age groups regarding seven geographical regions.

**Materials and methods:**

We included all patients from the Turkish population who received a first diagnosis of HF between January 1, 2016 and December 31, 2022, using ICD-10 codes from the National Electronic Healthcare Database. The data were categorized by seven geographical regions of Türkiye.

**Results:**

The median age of index diagnosis of HF was 70 (60–78) years in all age groups and 4 (1–12) years in pediatric population. The prevalence rate of HF was the highest in the Black Sea Region at 3.103%, while the Southeastern Anatolia Region exhibited the lowest at 1.436%. In all age groups, female patients with HF were older and had a higher prevalence rate across all geographical regions than male patients. From 2017 to 2021, incidence rates of HF declined to 3.0 per 1000 person years, with a consistent decrease for each geographical region. The highest incidence rates of HF were seen in the Black Sea Region, while the Southeastern Anatolia Region had the lowest. Evaluating pediatric population with HF, prevalence of HF was 0.81 per 1000 people (female children: 0.77 per 1000 people, male children: 0.84 per 1000 people). Female children with HF demonstrated the highest prevalence in the Central Anatolia Region with a rate of 1.04 per 1000 people, while male pediatric population with HF exhibited the greatest prevalence of HF in the Mediterranean Region, reaching 0.89 per 1000 people. The lowest prevalence of children with HF in both sexes was observed in the Eastern Anatolia Region (female children: 0.62 per 1000 people, male children: 0.48 per 1000 people).

**Conclusion:**

Despite regional variations, prevalence of HF in Türkiye’s regions aligns with global trends. Sex-based differences in HF prevalence were evident across all age groups, including pediatric population. Incidence rates of HF in each region exhibited a substantial decline by 2021.

## 1. Introduction

Heart failure (HF) is characterized as a global epidemic and is associated with severe morbidity and mortality. In an analysis conducted in the USA, the equivalent of 1.8% of the total population is estimated to be 5.7 million HF patients, with 870,000 new cases every year [1]. The worldwide total is projected to be 78 million in 2030 [2]. In all ages, the prevalence of HF was found to be 2.114% in Türkiye [3]. Additionally, it is important to note that there are also significant variations in its prevalence across different geographical regions [1]. Contemporary data from different countries indicated that the prevalence of HF is 1–3% in the general adult population [4]. The 2019 Heart Failure Association ATLAS Project reported the prevalence of HF ranging from ≤12 per 1000 people in Spain and Greece to >30 per 1000 people in Germany and Lithuania [5]. Finally, a study conducted by Şahin et al. showed in detail regional variations in the prevalence and incidence rates of adult patients with HF in Türkiye. The study revealed that the overall estimated prevalence of HF in adult patients with HF was 2.939%, exhibiting regional variations from 2.442% in the Southeastern Anatolia Region to 4.382% in the Black Sea Region [6].

The epidemiology of HF is well studied in adults with HF but obtaining accurate, high-quality data poses many challenges across all age groups affected by HF. The global epidemiology of HF in pediatric population has remained largely unknown. However, a prospective study in a pediatric tertiary center conducted by Massin et al. revealed that 10.4% of the 1196 pediatric patients diagnosed HF before the age of 16 [7]. In addition, more than 14,000 hospitalizations due to HF occur in pediatric population in the USA every year [8]. All these data originate exclusively from studies involving hospitalized children with HF within the same country and tertiary center. Consequently, a gap exists in contemporary epidemiological data regarding both ambulatory and hospitalized children with HF. This significant absence underscores the need for detailed investigation into the epidemiology of HF in pediatric populations.

Differences in access to HF treatments, quality of life, and mortality rates are evident between two sexes [9]. Additionally, the influence of socioeconomic deprivation specific to each country may exert a more pronounced in females than males [10]. Notably, the existing knowledge derives from studies conducted in high income countries, resulting in a lack of information regarding global sex differences in HF [10].

Overall, there has been a variation in prevalence rates of HF according to age, sex, and geographical region [1, 3, 6]. In addition, a greater understanding of the global epidemiology of the pediatric population with HF can lead to the development of a standardized definition for children as well as insights into etiologies, hospitalization rates, and HF costs. To address these knowledge gaps, we aimed to describe the epidemiologic data of HF in Türkiye across all age groups by sex in terms of geographical region between January 1, 2016 and December 31, 2022.

## 2. Materials and methods

This nationwide retrospective cohort study used deidentified data from the National Electronic Database of the Republic of Türkiye Ministry of Health. The database consists of all seven geographical regions, corresponding to a population of almost 85 million citizens. We included a total of 2,722,151 patients with HF who were citizens of the Republic of Türkiye in all age groups and admitted to public or private health institutions in Türkiye between January 1, 2016 and December 31, 2022. This study protocol was approved by the Republic of Türkiye Ministry of Health with the approval number 95741342-020. This study was conducted in accordance with the principles outlined in the Declaration of Helsinki. STROBE guidelines for cohort studies were used for the preparation of this report.

Patients with HF were obtained from the National Electronic Database with the use of specific International Statistical Classification of Diseases 10th revision (ICD-10) codes as follows: I50.0 (congestive HF), I50.1 (left ventricular dysfunction), I50.9 (HF, unspecified), I11.0 (hypertensive heart disease with congestive HF), I13.0 (hypertensive heart and chronic kidney disease with congestive HF), I13.2 (hypertensive heart and chronic kidney disease with congestive HF and renal failure), and I42.0 (dilated cardiomyopathy). Data on mortality were obtained from the Death Notification System of the General Directorate of Health Information Systems.

The prevalence rates of HF across all age groups and pediatric age groups were estimated by dividing the number of patients with HF who were alive at the end of 2022 by Türkiye’s population in 2022 for each geographical region. The annual incidence rates of HF across all age groups were calculated for each of the years from 2017 to 2022. The annual incidence rates were estimated by dividing the number of patients diagnosed with HF for the first time in a corresponding year by Türkiye’s population in that same year for each geographical region.

Geographical regions were classified according to ‘The First Geographical Congress in Ankara’ as follows: Marmara Region, Black Sea Region, Central Anatolia Region, Eastern Anatolia Region, Southeastern Anatolia Region, Mediterranean Region, Aegean Region.^1^

Data were divided into geographical regions, sex, and age. Categorical variables were presented as numbers (n) and frequencies (%). Continuous variables were presented as medians (interquartile range). SPSS 25.0 software program (IBM Corporation, Armonk, NY, USA) was used for all statistical analyses.

## 3. Results

The median age at index diagnosis of HF across all age groups was 70 (60–78) years. The median age of patients with HF in female and male was found to be 71 (62–80) and 68 (59–77) years, respectively. The Black Sea Region had the highest median age of HF for both sexes, while the Eastern Anatolia Region had the lowest median age of HF. The median age of HF for both females and males, categorized by geographical regions, was shown in Table 1.

In Türkiye, patients diagnosed with HF according to ICD-10 codes were distributed across various regions as follows: 30.45% in the Marmara Region, 15.83% in the Central Anatolia Region, 14.05% in the Aegean Region, 14.03% in the Black Sea Region, 11.97% in the Mediterranean Region, 7.07% in the Southeastern Anatolia Region, and 6.56% in the Eastern Anatolia Region. Table 2 showed the number of HF patients by sex across seven geographical regions.

Upon analyzing the population-adjusted prevalence of HF across regions, the Black Sea Region exhibited the highest prevalence at 3.103%, while the Southeastern Anatolia Region had the lowest at 1.436%. Sex-based prevalence analysis revealed a consistently higher population-adjusted prevalence in females across all geographical regions compared to males. Figures 1A and 1B illustrate that the highest population-adjusted incidence was seen in the Black Sea Region for both sexes and the lowest in the Southeastern Anatolia Region for both sexes.

**Figure 2:**
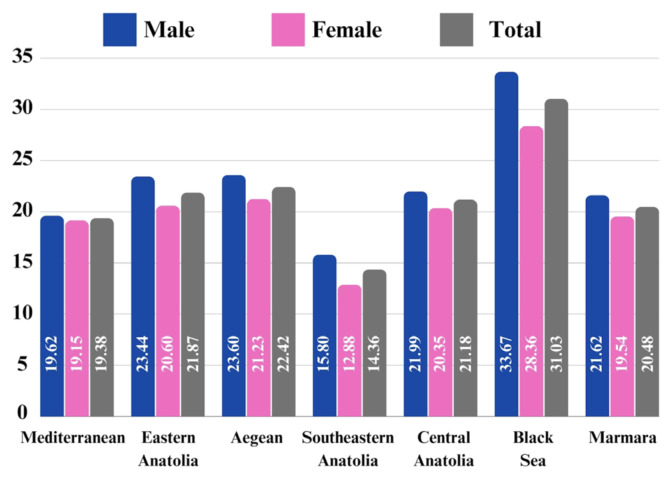
Prevalence rates of heart failure in all ages between 2017 and 2022 for each year according to seven geographical regions.

The Southeastern Anatolia Region had the lowest incidence rates of HF from 2017 to 2022, with this trend observed consistently each year. The Eastern Anatolia Region had the highest incidence rates of HF in both 2017 and 2018. Following the year 2018, the highest incidence rates of HF began to be seen in the Black Sea Region (Figures 1A and 1B). The prevalence rates of HF in Türkiye and in each geographical region were shown in Figure 2.

The median age of children with HF was 4 (1–12) years. The median ages of female and male children with HF were 3 (1–11) years and 4 (1–12) years, respectively (Table 3). Other median ages of children with HF for both sexes by seven distinct geographical regions were illustrated in Table 3.

The number of alive children with HF was estimated at 18,335; and the total population of children was 22,578,378 at the end of 2022. Therefore, the prevalence of children with HF in Türkiye was 0.81 per 1000 people (female children: 0.77 per 1000 people, male children: 0.84 per 1000 people) (Table 4). In female children with HF, the highest prevalence rate was seen in the Central Anatolia Region (1.04 per 100 people), while male children with HF had the highest prevalence rate in the Mediterranean Region (0.89 per 1000 people). The lowest prevalence of HF for both sexes was seen in the Eastern Anatolia Region (female children: 0.62 per 1000 people, male children: 0.48 per 1000 people) (Table 4).

## 4. Discussion

The present study investigated the consequences for HF patients across a wide spectrum of age (0–110 years) and the entire range of left ventricular ejection fraction in Türkiye and seven distinct geographical regions over a period of seven years. Overall, we observed significant variations in HF prevalence rates across geographical regions, with similar differences in incidence rates. The median age of HF was higher in female patients compared to their male counterparts in both Türkiye and all geographical regions. In contrast, when diagnosing HF, male child patients exhibited a higher median age than female child patients across the seven geographical regions. The prevalence rate of HF in children was found to be 0.81 per 1000 people in Türkiye, with varying prevalence rates for each sex in different geographical regions.

**Figure 3:**
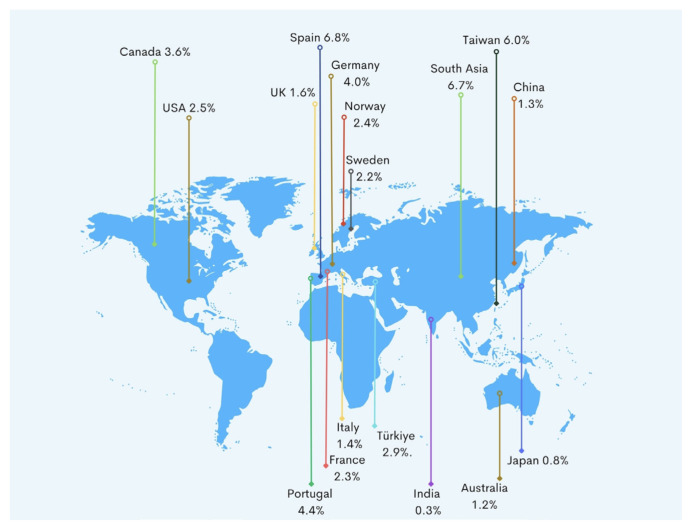
The comparison of prevalence of heart failure in several countries.

The prevalence rates of HF for each geographical region were found to be similar compared to other countries such as Western Europe, Canada, and the USA [5, 11, 12]. However, some Asian countries, including South America, China, Japan, Thailand, and South Korea, demonstrated lower prevalence rates than those observed in Türkiye’s regions (Figure 3) [13–15]. Germany emerged with higher prevalence rates of HF than each geographical region of Türkiye (Figure 3) [5]. The variability in prevalence rates of HF may stem from the underlying methodology employed in epidemiological trials, predominantly relying on data sourced from primary healthcare institutions and hospital registries [12]. Additionally, population-based analyses were also performed. In Switzerland and Türkiye, a population-based analyses using ICD-10 codes for HF revealed the prevalence rate of HF of 2.2% and 2.114%, respectively [3, 16]. In an analysis based on data for approximately fifty primary health care institutions in Belgium, the prevalence of HF was 1.2% in men and 1.3% in women. These rates notably fall below those observed in Switzerland and Türkiye [17].

The population-adjusted prevalence rates of HF displayed considerable variation across different geographical locations. Notably, the Black Sea Region exhibited the highest prevalence rate, while the Southeastern Anatolia Region had the lowest rate. This difference may be ascribed to the demographic composition of the Black Sea Region, where the population tends to have relatively higher ages compared to other regions [6].^2^ On the other hand, the Southeastern Anatolia Region’s lower prevalence of HF may be attributed to the region’s relatively constrained health resources. Factors such as a relatively lower number of doctors, nurses, midwives, and beds, coupled with decreased utilization of health services, could contribute to this discrepancy [18].

In the context of the incidence rate of HF, the highest incidence was observed in the Eastern Anatolia Region during the first two years, followed by a shift where the Black Sea Region took precedence from 2019 onwards, while the Southeastern Region recorded the lowest for seven years. This observed variation in incidence rates within Türkiye might be linked to a greater burden of cardiovascular risk factors in addition to a greater mean age in the Black Sea Region [6].^2^ This underscores the critical necessity for sustained and comprehensive community surveillance in diverse populations, particularly focusing on the prevalence and dynamics of cardiovascular risk factors. Across all geographical regions, a consistent trend of decreasing HF incidence rates was observed over the first 3-year study period. Within the nationwide electronic healthcare database, it is assumed that patients with HF might have received their initial HF diagnosis using ICD-10 codes for the first time during the year 2017. This assumption is based on the fact that 2016 was the year that mandatory nationwide registration started in Türkiye [19]. As a result, the estimated HF incidence rate in 2016 could potentially reflect a falsely high rate due to the transitional implementation of mandatory registration. Therefore, we think that from 2017 onwards, the HF incidence rate shows a more realistic state. Additionally, during the years 2020 and 2021, the incidence rates of HF in all geographical regions reached the lowest rate due to the COVID-19 pandemic [19]. Similarly, a nationwide cohort study from Denmark showed a decreasing pattern of new-onset HF during the COVID-19 pandemic [20].

In the pediatric HF cohort, the median age at the time of initial HF diagnosis was 4 (1–12) years. We observed the difference between both sexes according to the median age of index diagnosis of HF. Unlike adult patients with HF, in the pediatric population, the median age of index diagnosis of HF was higher in male children than female children, with a similar pattern for each geographical region. In the literature, the median age of index diagnosis of HF varied depending on the underlying etiology [21]. Specifically, children afflicted with congenital heart disease manifested a median age of 3 (0–11) months, while those diagnosed with cardiomyopathies or a combination of congenital heart disease and cardiomyopathies had a later presentation with median ages of 115 (17–185) months and 16 (2–133) months, respectively [21]. Overall, these findings may elucidate the temporal dynamics associated with HF onset in the pediatric population, underscoring the impact of underlying etiological factors.

In contrast to the epidemiological patterns observed in adult HF patients, the pediatric HF population in Türkiye showed a distinctive distribution, with the highest prevalence rate in the Central Anatolia Region. Remarkably, a similar finding was identified in the adult population, the lowest prevalence of HF among pediatric patients was found in the Eastern Anatolia Region. Our findings are consistent with a study by Kula et al., which showed that ventricular septal defect is the most common type of congenital heart disease in the pediatric population, particularly in the Central Anatolia Region [22]. Furthermore, as we know from the TRends-HF study, congenital heart disease was the most common comorbid condition in patients with HF under the age of 20 [3]. This concordance may reinforce the idea that the regional specificity of pediatric HF patterns highlights the important role of underlying specific etiologies in shaping epidemiological dynamics.

The main limitation of the study was the absence of data regarding the annual incidence rates of children with HF. We identified HF patients by using ICD-10 codes. However, ICD-10 codes do not indicate the phenotype of HF. Therefore, we lack specific information regarding the rate of HF phenotypes across the entire HF population.

## 5. Conclusion

This current study is important to show the epidemiological landscape of HF in the seven geographical regions of Türkiye across a wide age spectrum. The onset of HF manifested at different ages across seven distinct geographical regions within both the adult and pediatric populations. The incidence rates of HF in each geographical region exhibited a distinctive trend. Furthermore, the prevalence of HF showed a specific pattern, characterized by a persistent female predominance across diverse geographical regions in the adult population. Conversely, within the pediatric population with HF, there was a notable prevalence of male dominance.

## Figures and Tables

**Figure 1A:**
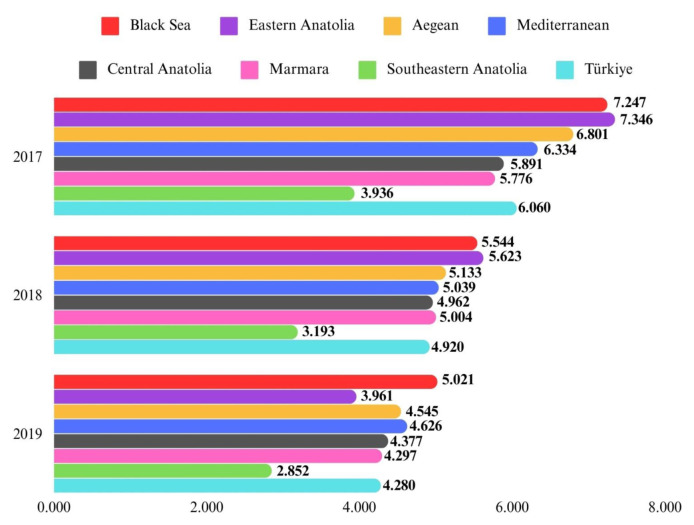
Population-adjusted incidence of heart failure in all ages by sex across seven geographical regions between 2017 and 2019.

**Figure 1B:**
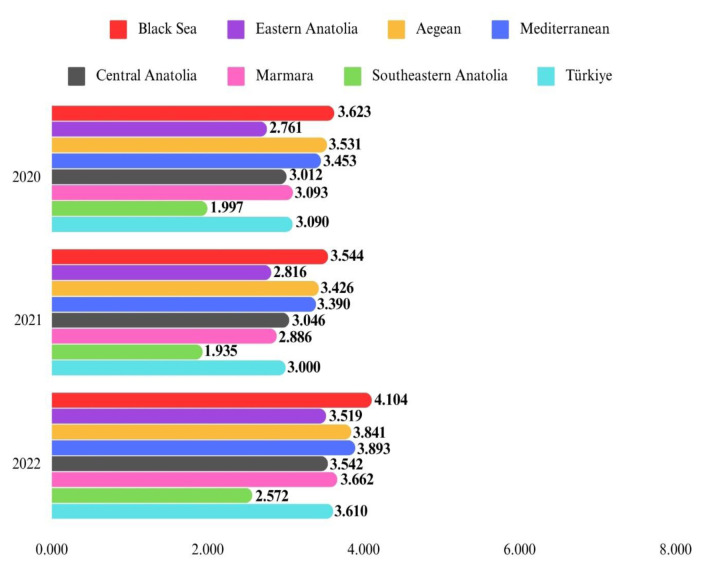
Population-adjusted incidence of heart failure in all ages by sex across seven geographical regions between 2020 and 2022.

**Table 1:** Median age of heart failure in all ages by sex across seven geographical regions.

	Female (n: 1,407,927)	Male (n: 1,314,224)	Total (n: 2,722,151)
**Türkiye (years)**	71 (62–80)	68 (59–77)	70 (60–78)
**Marmara Region (years)**	72 (62–80)	67 (58–76)	70 (60–78)
**Central Anatolia Region (years)**	71 (62–79)	68 (59–77)	70 (61–78)
**Aegean Region (years)**	72 (63–81)	69 (60–77)	71 (61–79)
**Black Sea Region (years)**	73 (64–81)	70 (61–78)	71 (63–79)
**Mediterranean Region (years)**	71 (62–80)	67 (58–76)	69 (60–78)
**Southeastern Region (years)**	69 (58–77)	66 (56–75)	67 (57–76)
**Eastern Region (years)**	68 (58–77)	66 (57–75)	67 (58–76)

**Table 2:** Number of patients with heart failure in all ages by sex across seven geographical regions.

	Female (n: 1,407,927)	Male (n: 1,314,224)	Total (n: 2,722,151)
**Marmara Region n, (%)**	428,442 (30.4)	400,521 (30.5)	828,963 (30.5)
**Central Anatolia Region n, (%)**	220,554 (15.7)	210,428 (16.0)	430,982 (15.8)
**Aegean Region n, (%)**	198,021 (14.1)	184,704 (14.1)	382,725 (14.1)
**Black Sea Region n, (%)**	203,018 (14.4)	179,064 (13.6)	382,082 (14.0)
**Mediterranean Region n, (%)**	163,264 (11.6)	162,828 (12.4)	326,092 (12.0)
**Southeastern Region n, (%)**	102,787 (7.3)	89,758 (6.8)	192,545 (7.1)
**Eastern Region n, (%)**	91,841 (6.5)	86,921 (6.6)	178,762 (6.6)

**Table 3:** Median age of heart failure in children by sex across seven geographical regions.

	Female children (n: 9872)	Male children (n: 11,180)	Total children (n: 21,052)
**Türkiye (years)**	3 (1–11)	4 (1–12)	4 (1–12)
**Marmara Region (years)**	3 (1–11)	4 (1–12)	3 (1–11)
**Central Anatolia Region (years)**	4 (1–12)	6 (1–13)	4 (1–12)
**Aegean Region (years)**	5 (1–13)	7 (1–14)	6 (1–13)
**Black Sea Region (years)**	5 (1–13)	6 (1–13)	6 (1–13)
**Mediterranean Region (years)**	2 (1–10)	3 (1–11)	3 (1–11)
**Southeastern Anatolia Region (years)**	3 (1–10)	3 (1–11)	3 (1–10)
**Eastern Anatolia Region (years)**	3 (1–12)	3 (1–12)	3 (1–12)

**Table 4:** The prevalence rates (per 1000 people) of children with heart failure by sex across seven geographical regions.

	Female children (n: 9872)	Male children (n: 11,180)	Total children (n: 21,052)
**Türkiye n, (%)**	0.77	0.84	0.81
**Marmara Region n, (%)**	0.72	0.83	0.78
**Central Anatolia Region n, (%)**	1.04	0.86	0.94
**Aegean Region n, (%)**	0.76	0.84	0.80
**Black Sea Region n, (%)**	0.65	0.65	0.65
**Mediterranean Region n, (%**)	0.89	0.89	0.89
**Southeastern Anatolia Region n, (%)**	0.86	0.75	0.81
**Eastern Anatolia Region n, (%)**	0.62	0.48	0.54

## Footnotes

1 The first geographical congress in Ankara (Türkiye) [online]. Website https://tr.wikipedia.org/wiki/T%C3%BCrkiye%27nin_co%C4%9Frafi_b%C3%B6lgeleri [accessed 13 April 2024].

2 Yaşlı Nüfus İstatistik Bülteni (2020). Aile ve Sosyal Hizmetler Bakanlığı, Engelli ve Yaşlı Hizmetleri Genel Müdürlüğü [online]. Website https://www.aile.gov.tr/eyhgm/haberler/2020-yili-yasli-nufus-istatistik-bulteni-yayinlandi/ [accessed 05 October 2021].
